# Cryo-Electrospinning Generates Highly Porous Fiber Scaffolds Which Improves Trabecular Meshwork Cell Infiltration

**DOI:** 10.3390/jfb14100490

**Published:** 2023-09-22

**Authors:** Devon J. Crouch, Carl M. Sheridan, Julia G. Behnsen, Raechelle A. D’Sa, Lucy A. Bosworth

**Affiliations:** 1 Department of Eye and Vision Science, Institute of Life Course and Medical Sciences, Faculty of Health and Life Sciences, University of Liverpool, Liverpool L7 8TX, UK; d.crouch@liverpool.ac.uk (D.J.C.); carlos@liverpool.ac.uk (C.M.S.); 2 Department of Mechanical, Materials, and Aerospace Engineering, University of Liverpool, Liverpool L69 6GB, UK; julia.behnsen@liverpool.ac.uk; 3 School of Engineering, University of Liverpool, Liverpool L69 3GH, UK; rdsa@liverpool.ac.uk

**Keywords:** electrospinning, cryogenic electrospinning, trabecular meshwork, porosity, pore size, cell infiltration, cell attachment, three dimensional, biomimicry, polycaprolactone

## Abstract

Human trabecular meshwork is a sieve-like tissue with large pores, which plays a vital role in aqueous humor outflow. Dysfunction of this tissue can occur, which leads to glaucoma and permanent vision loss. Replacement of trabecular meshwork with a tissue-engineered device is the ultimate objective. This study aimed to create a biomimetic structure of trabecular meshwork using electrospinning. Conventional electrospinning was compared to cryogenic electrospinning, the latter being an adaptation of conventional electrospinning whereby dry ice is incorporated in the fiber collector system. The dry ice causes ice crystals to form in-between the fibers, increasing the inter-fiber spacing, which is retained following sublimation. Structural characterization demonstrated cryo-scaffolds to have closer recapitulation of the trabecular meshwork, in terms of pore size, porosity, and thickness. The attachment of a healthy, human trabecular meshwork cell line (NTM_5_) to the scaffold was not influenced by the fabrication method. The main objective was to assess cell infiltration. Cryo-scaffolds supported cell penetration deep within their structure after seven days, whereas cells remained on the outer surface for conventional scaffolds. This study demonstrates the suitability of cryogenic electrospinning for the close recapitulation of trabecular meshwork and its potential as a 3D in vitro model and, in time, a tissue-engineered device.

## 1. Introduction

The use of electrospinning has increased rapidly over recent decades because it is a simple, cost-effective, and versatile method for fabrication of fiber scaffolds that may be broadly applied in a range of applications, including, but not limited to, tissue engineering [1], wound dressing [2], drug delivery [3], sensors [4], hygiene systems [5], and filtration [6]. Electrospun fiber scaffolds are widely researched in the tissue engineering field due to their high-surface-area-to-volume ratio and ability to mimic the host tissue’s extracellular matrix (ECM) and support cell attachment and growth [7]. As such, electrospinning has been utilized to mimic a variety of different human tissues, including bone [8], cardiac [9], skin [10], and ocular [11]. However, electrospun fibers can be limited in their usefulness for certain tissue engineering applications as cell movement into the structure can be hindered by the dense network of fibers and a small pore size [12], which therefore prevents these scaffolds from truly imitating the host tissue and facilitating their native cellular activity. This often leads to electrospun scaffolds being considered as two-dimensional (2D) or 2.5D structures rather than three-dimensional (3D) [13,14,15,16].

The ability to better control the pore size could allow a more accurate mimicry of natural tissue spacing to be achieved, which may also allow cells to interact with these synthetic scaffolds more optimally [17,18]. Varying the polymeric solution properties (e.g., viscosity, volatility, conductivity) and electrospinning parameters (e.g., applied voltage, flow rate) is known to directly affect the final fiber diameter and the inter-fiber spacing (i.e., pore size) [19]. Other approaches include sacrificial fiber and microparticle spinning [20,21]. The former involves two polymers electrospun in parallel to create a dual fiber scaffold where one polymer is subsequently dissolved in solution (e.g., polyethylene oxide in water) to leave just one type of polymeric fiber [20]. The latter has a very similar approach, but the sacrificial component is simultaneously electrosprayed to incorporate water-soluble microparticles (e.g., polyethylene oxide) amongst the fibers that are later washed out [21]. Both sacrificial techniques are successful in creating fiber scaffolds with larger inter-fiber spacing; however, extra costs in equipment set-up and materials are required.

**Figure 1:**
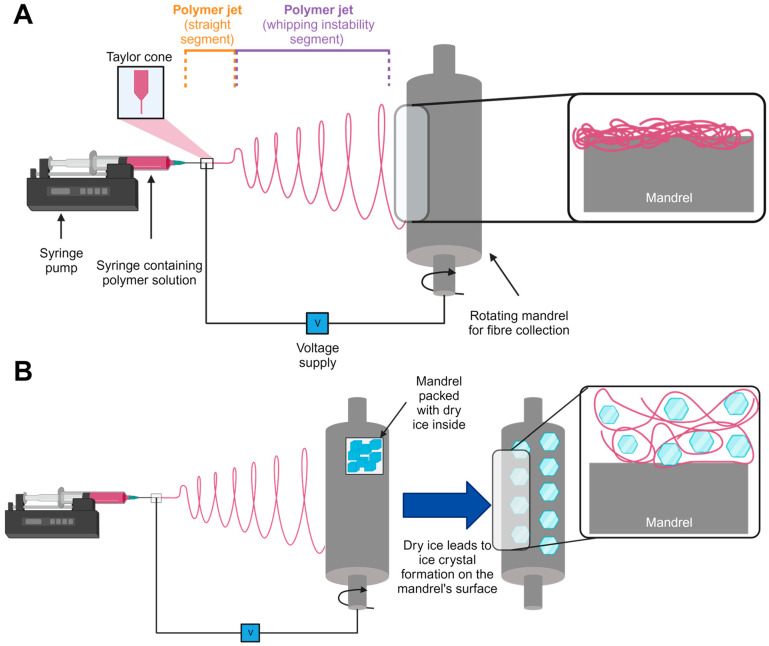
**Electrospinning and cryogenic electrospinning processes.** Schematic illustrating (**A**) conventional electrospinning and (**B**) cryogenic electrospinning set-ups.

Wet electrospinning has also been reported as a viable approach to increase pore size [22]. Here, electrospun fibers penetrate a liquid coagulation bath and are held suspended within the liquid to create a porous, 3D structure. Whilst advantageous, this technique requires a vertical electrospinning set-up, which may not be appropriate for all systems [23]. Another method to increase pore size and overall porosity, which is not dependent on equipment orientation and requires just the polymer of interest, is cryogenic electrospinning (cryo-spinning) [24]. This technique utilizes a collector system packed with dry ice to enable ice crystal formation during fiber collection, which spaces the fibers further apart, and this inter-fiber spacing is retained following sublimation (Figure 1).

Cryo-spun fiber scaffolds have been researched and developed for mimicry of several different tissues, including vasculature [25], kidney [26], and cartilage [27]. Another tissue where cryo-spinning could be suitable is the trabecular meshwork (TM). The TM is a hierarchically porous tissue located in the anterior segment of the eye and is responsible for regulating the outflow of aqueous humor, and, in turn, intraocular pressure [28,29]. This tissue comprises a chaotic arrangement of collagenous beams resulting in a complex pore structure ranging between 8.3 and 12.8 µm in their diameter and an overall porosity of 85.0 ± 1.0% [30]. The literature suggests that TM cells are directly influenced by the size of pores they are grown on by exhibiting increased proliferation and greater quantities of fibronectin deposition when cultured on larger pores in vitro [18].

In this study, poly(ε-caprolactone) (PCL) was selected as an exemplar polymer because it is commonly used for tissue engineering applications owing to its biocompatibility, biodegradability, and ease of tailoring electrospinning parameters to create a broad range of fiber scaffolds [31,32]. Here, we aim to determine the utility of cryo-electrospun scaffolds compared to conventional electrospun scaffolds as a mimic for trabecular meshwork, in terms of their structural and mechanical properties and capability to support TM cell infiltration. We hypothesize cryo-spinning PCL will fabricate a highly porous electrospun scaffold that better mimics the TM’s structure and supports greater TM cell infiltration compared to conventionally electrospun PCL.

## 2. Materials and Methods

### 2.1. Solution Preparation

Poly(ε-caprolactone) with inherent viscosity dL/g = 1.2 (molecular weight measured in-house using gel permeation chromatography as M_n_ = 70,000 g/mol (data not included)) (PCL; Purasorb PC12, Corbion, Amsterdam, The Netherlands) was dissolved in 1,1,1,3,3,3-hexafluorisoproponal (HFIP; Sigma-Aldrich, Gillingham, UK) at 12% weight/volume (*w*/*v*) and stirred continuously at room temperature for 48 h until complete dissolution. Then, 0.001% *w*/*v* rhodamine (Sigma-Aldrich, Gillingham, UK) was added prior to electrospinning.

### 2.2. Scaffold Fabrication

Electrospinning was conducted using an IME Technologies EC-CLI unit with a controlled ambient environment (temperature 23.0 °C, humidity 50%). The polymer solution was loaded into a capillary-ended syringe with a flow rate of 1 mL/h. Other spinning parameters included: applied voltage +15 kV, collector voltage −4 kV, needle-to-collector distance 17 cm, and hollow collector mandrel (length = 180 mm, diameter = 90 mm) rotating at 100 RPM. Lateral movement of the capillary during fiber collection included motion settings of distance 125 mm, speed 5 mm/s (translational speed), and dwell time 5 mm/s. Total spin time for all runs was 50 min. Emitted fibers were collected on a mandrel covered with wax paper (PME, Enfield, UK) (N = 3).

The only variable was the set-up of the mandrel used to collect fibers during each electrospinning run. Standard electrospun fibers of PCL were collected using the mandrel in its regular set-up (termed ‘PCL’). The other electrospun PCL scaffold was fabricated using a mandrel packed with dry ice, which caused ice crystals to form on the surface of the mandrel during fiber deposition (termed ‘cryo-PCL’). Collected cryo-PCL fibers were immediately placed in a vacuum desiccator overnight to allow for complete water removal without structural collapse (N = 3).

Both types of scaffolds were removed from the wax backing paper using forceps without any noticeable difference between the groups in terms of their handling prior to characterization.

### 2.3. Material Characterisation

#### 2.3.1. Fiber Morphology and Diameter Size

Scanning electron microscopy (SEM) was used to obtain high-magnification images of fabricated PCL- and cryo-PCL-electrospun scaffolds. Scaffolds were mounted on carbon-tabbed SEM stubs (Agar Scientific Ltd., Stansted, UK) and gold/palladium (AuPd) sputter-coated (Quorum 1050T S, Quorum Technologies, East Sussex, UK) to increase electrical conductivity. Scaffolds were imaged using a HITACHI TM4000 Plus tabletop SEM at high vacuum with 15 kV electron beam. Two distinct areas of each fiber sheet were imaged per experimental repeat.

Fiber diameters were measured from SEM images using Fiji ImageJ software (v.2.0.0/1.53c, National Institutes of Health, Bethesda, MD, USA) (n = 100). This was achieved by using the scale bar of each image to initially set the scale followed by the line draw tool. Fiber diameters were statistically analyzed using GraphPad Prism v9.1.0 (San Diego, CA, USA). The data were not normally distributed (Shapiro–Wilks normality test) and were subsequently analyzed using a two-tailed Mann–Whitney test. The data were presented as median and interquartile (IQR) range.

#### 2.3.2. Fluorescent Imaging

PCL and cryo-PCL fiber scaffolds were mounted and secured to a glass slide (VWR, Lutterworth, UK) using a glass coverslip (VWR, Lutterworth, UK). Z-stack images of rhodamine-stained fiber scaffolds were collected using an LSM 800 confocal microscope (Carl Zeiss Microscopy Ltd., Cambridge, UK). Z-stack images were taken using a 20× objective, and each image slice represented 1.46 µm. Images were processed using ImageJ and displayed as a singular slice through the central region of the scaffold to highlight the difference in their porosities.

#### 2.3.3. X-ray Computed Tomography and Structural Analysis

X-ray computed tomography (X-CT) scans of PCL and cryo-PCL scaffolds were acquired using a Zeiss Xradia Versa 620 instrument. The 40× objective was selected, and source and detector distances were set to achieve a voxel size of 1.08 µm, which resulted in a 350 µm × 350 µm field of view (detector binning 2). A source accelerating voltage of 60 kV at 6.5 W was applied. No beam filter was used. For each scan, 3201 projections were acquired over 360 ° with an exposure time of 3 s per projection. The projection data were reconstructed using Zeiss proprietary Reconstructor Scout-and-Scan, v16.1.13038, which employs a cone beam Feldkamp–Davis–Kress algorithm. Then, 3D images of the reconstructed data were created using Drishti 2.7 [33].

Original reconstructed data files from the X-CT imaging of electrospun scaffolds (.TXM file) were analyzed in ImageJ using the BoneJ plugin to allow measurement of the average pore diameter and overall porosity [34,35]. Data for Trabecular Spacing using BoneJ plugin were collected as mean ± standard deviation (Mean ± SD). The porosity of the scaffolds was calculated using the BoneJ feature ‘Area/Volume Fraction’, which results in three measurements: the BV (mm^3^; space occupied by fibers); the TV (mm^3^; total volume of scaffold); and the Area/Volume Fraction (BV/TV). From this, the open volume (OV; mm^3^; unoccupied space (pore space)) could be calculated by:

TV − BV = OV, (1)

OV/TV × 100 = porosity (%) (2)

Scaffold thickness was measured using the ImageJ measure tool (n = 30).

Data collected were normally distributed (Shapiro–Wilk normality test). Therefore, the statistical analysis involved two-tailed unpaired t-tests using GraphPad Prism v.9.1.0.

#### 2.3.4. Tensile Testing

Using a scalpel, fiber scaffolds were cut into 30 × 10 mm rectangles and secured over a paper window (using sticky tape) to give final dimensions of 20 × 10 mm. Paper windows were individually positioned within the tensile grips and the sides cut before the test was started to ensure only the scaffold was loaded.

A UniVert (CellScale; Waterloo, ON, Canada) in tensile mode with 1 N load cell and strain rate of 10% strain/min (n = 12) was used. The data were processed in GraphPad Prism v.9.1.0. A two-tailed unpaired t-test was applied (GraphPad Prism v.9.1.0) as the data were normally distributed (Shapiro–Wilk normality test). The data were presented as Mean ± SD.

### 2.4. In Vitro Cell Culture

#### 2.4.1. Preparation of Cell Culture Samples and Cell Seeding

A transformed human normal trabecular meshwork 5 (NTM_5_) cell line was obtained from Alcon Research Laboratories (Fort Worth, TX, USA) and was cultured at 37 °C and 5% CO_2_ in 75 cm^2^ sterile flasks (Greiner Bio-one, Stonehouse, UK) with Dulbecco’s modified Eagle’s medium (DMEM; Gibco™, Loughborough, UK), low-glucose medium supplemented with 10% *v/v*, fetal bovine serum (FBS; Labtech, East Sussex, UK), 1% *v/v* penicillin (Merck, Gillingham, UK), and 1% *v/v* streptomycin (Merck, Gillingham, UK).

PCL and cryo-PCL scaffolds were held within CellCrown™ 24NX (Scaffdex Oy, Tampere, Finland). Samples were sterilized by ultraviolet light exposure for 15 min followed by immersion in 70% *v/v* ethanol (VWR, Lutterworth, UK) for 8 h. Samples were subsequently washed thrice with phosphate-buffered saline solution (PBS; Thermo Fisher, Loughborough, UK), placed in sterile low binding 24-well plates (Corning, Glendale, Arizona, USA), and immersed in DMEM for 24 h to allow protein adsorption on to the surface of the fibers.

NTM_5_ cells (60,000 cells/cm^2^) were seeded onto PCL, cryo-PCL, and culture-treated 24-well plates (Corning, UK) and low binding 24-well plates (n = 6). Seeded cells were left for 30 min to allow cells to form initial attachments. Wells each received an additional 1 mL of DMEM low glucose media and were subsequently cultured for a total period of 7 days at 37 °C and 5% CO_2_.

#### 2.4.2. Cell attachment assessment

Attachment of NTM_5_ cells on the scaffolds after a 4 h culture period was determined by PicoGreen™ assay (Fisher Scientific, Loughborough, UK). The total DNA content of one sample was split into three fractions: scaffold, media, and well, which was subsequently used to determine the percentage of cells attaching to the sample. Both 2D controls (positive culture-treated well plate; negative low-binding well plate) did not contain a scaffold; therefore, only media and well fractions were measured.

The assay buffer (lysis) was prepared by addition of Triton X-100 (1%) into 1× TE buffer (0.5 mM Tris-HCL, 0.05 mM EDTA). A stock solution of PicoGreen (0.5%) in 1× TE buffer was made. All culture media were removed from samples and placed in appropriately labelled sterile microfuge tubes, where they were centrifuged at 1000 RPM for 5 min. The supernatant was removed, and 1 mL assay buffer was added; this constitutes the media fraction of the samples. The PCL and cryo-PCL samples were separately washed with PBS and added into sterile microfuge tubes each containing 1 mL of assay buffer (scaffold fraction). The well fraction was obtained by adding 1 mL assay buffer to the vacant well after scaffold and media removal. The bottom of the well was thoroughly scraped with a pipette tip and the buffer subsequently removed from the well and placed into a sterile microfuge tube. All microfuge tubes were vortexed for 20 s and stored at −80 °C.

The PicoGreen assay involved thawing all samples to room temperature and subsequently aliquoting 100 µL from each sample (in duplicate) and placing into a black 96-well plate (Corning, UK). Then, 100 µL of PicoGreen working solution was added to each sample, homogenized, and then fluorescence-measured at a 520 nm emission on a multi-plate reader (FLUOstar Omega; Isogen Life Science, Utrecht, The Netherlands).

Absorbance units provided from the multi-plate reader for each data set were converted into a cell number by using an NTM_5_ cell line standard curve (not included). Data were analyzed using GraphPad Prism V.9.1.0 and presented as Mean ± SD.

#### 2.4.3. Cell infiltration Analysis

Cell movement within each of the electrospun structures after 7 days was observed through confocal microscopy (ZEISS LSM 800, Oberkochen, Germany). Aliquots of NTM_5_ cells (60,000 cells/cm^2^) were seeded onto the scaffolds (n = 3). Cells were left for 30 min to allow adhesion before adding 1 mL of DMEM low glucose and incubated for 7 days, with media changes every 3 days. After the culture period had elapsed, media were removed from all samples, washed twice with PBS, and fixed in 10% *v/v* neutral buffered formalin (NBF; Sigma-Aldrich, Gillingham, UK) for 15 min. NBF was removed, samples washed thrice with PBS, and samples then permeabilized with Triton X-100 (0.5% *v*/*v*) in PBS for 15 min. Scaffolds were stained for cell cytoskeleton by immersing them in phalloidin (1% *v/v* (Invitrogen, Loughborough, UK) in 1% bovine serum albumin in PBS) at 37 °C for 1 h. After incubation, the phalloidin solution was removed and substrates washed thrice with PBST (PBS + 0.1% tween). Cell nuclei were stained with 0.01% *v/v* DAPI (4′,6-diamidino-2-pheny-lindole; Sigma-Aldrich, Gillingham, UK) in PBS for 20 min at room temperature. Scaffolds were washed in PBS and mounted onto glass slides for imaging.

Orthogonal slices (1.46 µm thickness) were used to view cell infiltration into the scaffold. Depth of migration was determined by measuring the top of the rhodamine-stained scaffold down to the midpoint of a DAPI-stained cell (N = 3, n = 10; Fiji ImageJ v.2.0.0/1.53c).

## 3. Results

### 3.1. Scaffold fabrication and analysis

A 12% *w/v* solution of PCL was successfully electrospun using pre-determined spinning parameters and set spin time. The only variable was the method of fiber collection: standard electrospinning using a slowly rotating mandrel versus cryo-electrospinning, where the mandrel rotates at the same speed but is packed inside with dry ice. The purpose of incorporating dry ice is to encourage ice crystal formation on the surface of the mandrel, which then interferes with fiber packing upon collection. Separate 50 min runs resulted in the fabrication of electrospun fiber sheets that were subsequently analyzed to compare any structural differences between these two experimental groups.

#### 3.1.1. SEM and Confocal 2D Imaging

**Figure 2:**
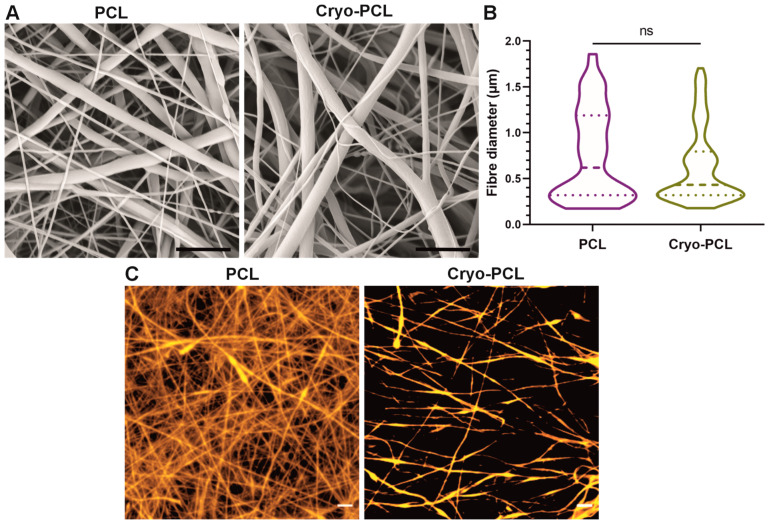
**Cryogenic electrospinning increased spacing between fibers without affecting morphology or diameter.** (**A**) Scanning electron micrographs of electrospun poly(ε-caprolactone) (PCL) and cryogenic electrospun PCL (cryo-PCL) (magnification ×10,000, scale bar = 5 µm). (**B**) Fiber diameters displayed as a violin plot demonstrating fiber diameter distribution, median, and interquartile range for PCL and cryo-PCL (n = 100). Two-tailed Mann–Whitney statistical test (*p* < 0.05), with non-significance represented as ns. (**C**) Confocal singular slice images (thickness 1.46 µm) of PCL and cryo-PCL (orange = rhodamine-stained fibers) (objective 20×, scale bar = 10 µm).

SEM images for both fabrication conditions presented no visual differences, with fibers appearing uniform, bead-free and, due to the slow mandrel rotation speed, randomly orientated. Furthermore, surface morphologies also appeared unchanged, displaying seemingly smooth surfaces for both experimental groups (Figure 2A). Fiber spacing appeared to be greater for the cryo-spun fibers, but this could not be determined by this imaging technique. The measurement of fiber diameter demonstrated no significant change between the two groups (Figure 2B). The median fiber diameter for standard PCL was 0.6 µm (IQR 0.32–1.19µm), and for cryo-PCL, 0.43 µm (0.32–0.80 µm).

Confocal microscopy of the rhodamine-stained fibers, however, demonstrated a clear difference in fiber packing, and hence pore size, between PCL and cryo-PCL (Figure 2C). Standard PCL fibers revealed a densely packed fibrous network with small pore sizes, whereas fibers collected in the presence of dry ice presented a more open structure with larger pore sizes.

#### 3.1.2. X-CT 3D Imaging and Structural Analysis

**Figure 3:**
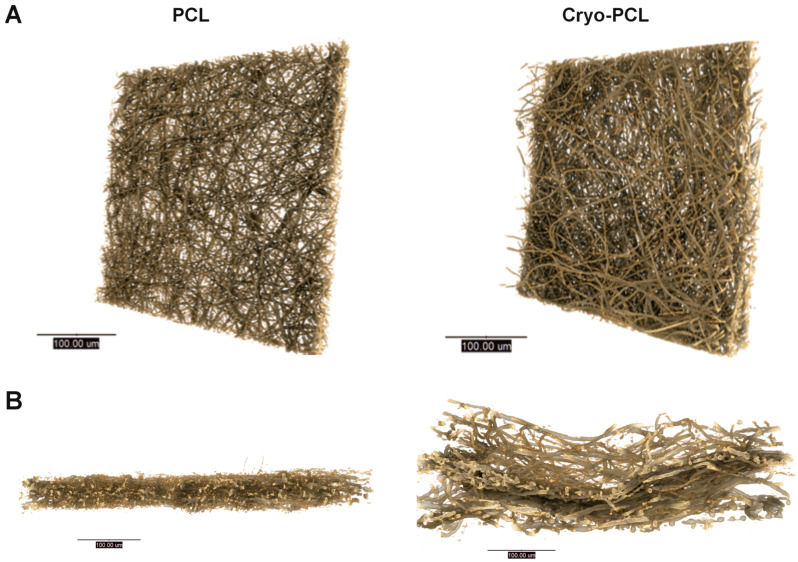
**Cryogenic electrospinning increases pore size and thickness of electrospun PCL scaffolds.** X-ray computed tomography images of electrospun poly(ε-caprolactone) (PCL) and cryo-electrospun PCL (cryo-PCL) presented in (**A**) front-on and (**B**) cross-sectional orientations (scale bars = 100 µm).

X-CT imaging enabled three-dimensional (3D) visualization of the electrospun scaffolds (Figure 3). The top view of the scaffolds revealed no evident difference in structure between PCL and cryo-PCL (Figure 3A). Cross-sectional views of PCL and cryo-PCL demonstrated an obvious difference in scaffold porosity and thickness (Figure 3B), where cryo-PCL scaffolds revealed a thicker structure with greater inter-fiber spacing compared to the standard PCL, which appeared thin and densely packed.

Structural analysis from these X-CT 3D images were obtained for both PCL and cryo-PCL (Table 1), which highlighted significant increases in pore size (3.3 ± 0.8 µm to 8.5 ± 2.0 µm; *p* < 0.0144), porosity (70.9 ± 3.8% to 91.9 ± 3.4%; *p* < 0.0021), and scaffold thickness (30.4 ± 2.9 µm to 76.9 ± 10.8 µm; *p* < 0.0001) for cryo-PCL scaffolds compared to standard PCL scaffolds.

#### 3.1.3. Tensile Testing

**Figure 4:**
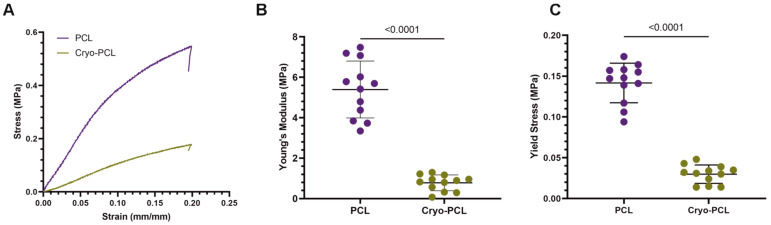
**Cryo-spinning significantly reduces the tensile properties of the fiber scaffold.** (**A**) Typical stress–strain curve, (**B**) Young’s modulus, and (**C**) yield stress of electrospun poly(ε-caprolactone) (PCL) and cryo-electrospun PCL (cryo-PCL). Data presented as mean ± standard deviation (n = 12). Two-tailed unpaired *t*-test statistical test. Statistical differences presented by *p* values with *p* < 0.05 considered significant.

PCL and cryo-PCL scaffolds were tensile-tested to determine the impact of scaffold manufacture on mechanical properties. Figure 4A presents a typical stress–strain curve for the tensile testing of PCL and cryo-PCL. Scaffold stiffness was directly affected by its structure: standard PCL fibers yielded a Young’s modulus of 5.15 ± 0.55 MPa, which was significantly reduced for cryo-PCL fibers (0.79 ± 0.24 MPa; *p* < 0.0001) (Figure 4B). A similar observation occurred when measuring the tensile strength of the scaffolds. At the point of elastic-to-plastic deformation, standard PCL fibers yielded a strength of 0.17 ± 0.02 MPa compared to 0.03 ± 0.01 MPa for cryo-PCL fibers (*p* < 0.0001) (Figure 4C).

### 3.2. In Vitro Response

#### 3.2.1. Cell Attachment

**Figure 5:**
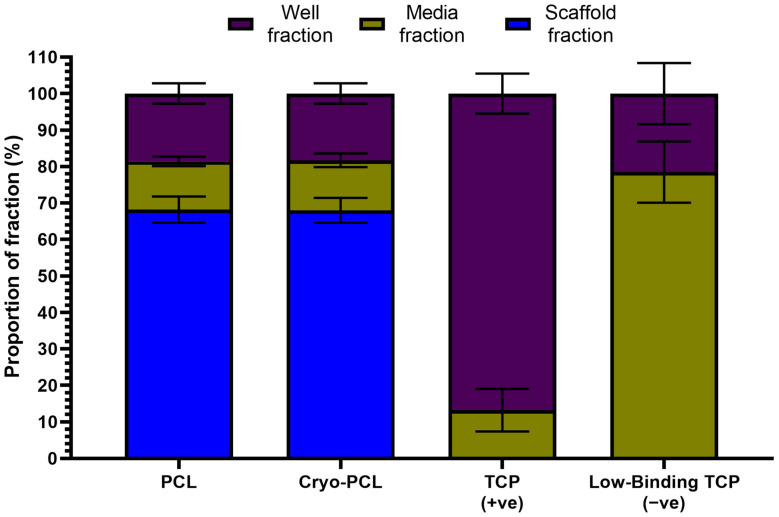
**Cryogenic electrospinning did not affect initial cell attachment.** Percentage of normal trabecular meshwork (NTM_5_) cell attachment to electrospun poly(ε-caprolactone) (PCL), cryo-electrospun PCL (cryo-PCL), tissue culture-treated well plates (TCP; positive control), and low-binding TCP well plates (negative control) (n = 6). Cell location split across three different fractions: adherent to the scaffold or the well, or circulating in the media. Note: no scaffold was present in either control group. Data presented as mean ± standard deviation.

PicoGreen binds and fluoresces dsDNA and, in conjunction with a standard curve (not shown), can be used to determine the number of cells present within a sample. The initial cell attachment of NTM_5_ cells to the scaffolds after a period of 4 h was assessed, split into fractions (scaffold, media, and well), and displayed as a total percentage of 100 (Figure 5). Data analysis revealed that 68.2 ± 3.6% of cells were attached to the electrospun PCL fibers, with 13.2 ± 1.3% floating freely in the media and 18.6 ± 2.8% adherent to the base of the well. The same trend was observed for the cryo-PCL scaffold (68.0 ± 3.4% scaffold, 13.7 ± 1.9% media, and 18.3 ± 2.8% well). The majority of cells adhered to the base of the well for the positive control (86.8 ± 5.5% well; 13.2 ± 5.8% media), and the opposite was observed for the negative control (78.5 ± 8.4% well; 21.5 ± 8.4% media).

#### 3.2.2. Cell Infiltration

**Figure 6:**
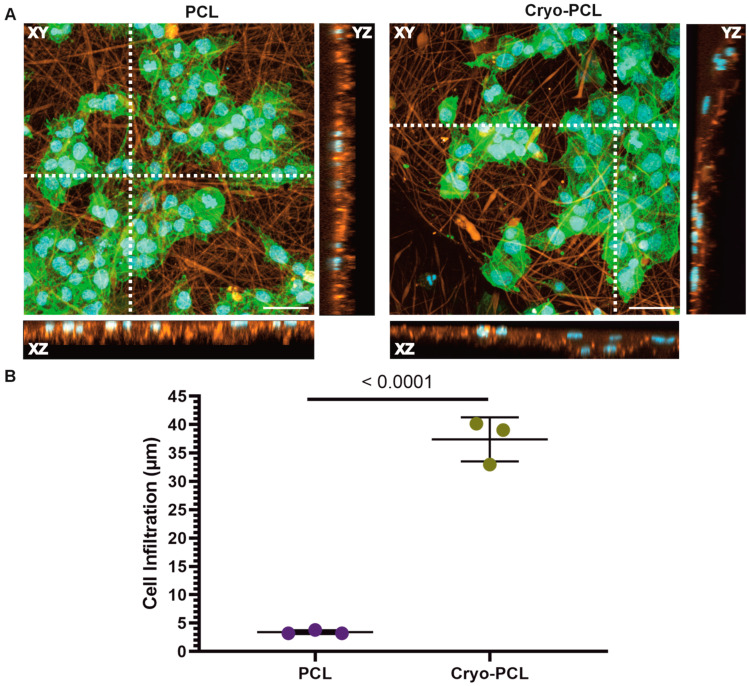
**Cryogenic electrospinning facilitated increased cell infiltration within the scaffold.** (**A**) Representative confocal z-stacked images of NTM_5_ cells after 7 days in culture on electrospun poly(ε-caprolactone) (PCL) scaffolds and cryo-electrospun PCL (cryo-PCL). Immunocytochemistry staining of cell nucleus (DAPI, blue), cell cytoskeleton (phalloidin-488, green), and fibers (rhodamine, orange). Images shown: XY z-stack (20× objective, scale bar = 50 µm), XZ, and YZ side view. White dashed line on XY z-stack represent position of XZ (horizontal) and YZ (vertical) images. (**B**) Quantification of NTM_5_ cell infiltration into both PCL and cryo-PCL scaffolds after 7 days in culture. Data presented as mean ± standard deviation, where each data point represents the mean from 10 measurements of an individual sample. Two-way ANOVA statistical test. Statistical differences presented by *p* values (*p* < 0.05).

The addition of rhodamine prior to electrospinning allowed cell location relative to the now-fluorescent fibers in the 3D scaffold to be observed. Scaffolds were imaged after seven days in culture to determine if NTM_5_ cells had penetrated the structures. Figure 6A displays representative XY z-stacks and XZ/YZ side views. For both scaffolds, the XY images displayed a reasonable cell coverage of the scaffolds’ surface after seven days. The XZ and YZ profiles revealed further information regarding the placement of cells, with cells remaining on the upper surface of the PCL scaffold but with evidence of cells within the scaffold structure for the cryo-PCL. The depth of each cell nucleus relative to the upper surface for both scaffold types was measured (Figure 6B). Cryo-PCL facilitated cell penetration up to a depth of 37.39 ± 6.13 µm compared to PCL, where cells only reached a distance of 3.41 ± 1.17 µm (*p* < 0.0001).

## 4. Discussion

This paper investigated the impact of incorporating dry ice during electrospinning on fiber formation, scaffold arrangement, and subsequent infiltration of a healthy, human trabecular meshwork cell line, NTM_5_, into the structure.

Using poly(ε-caprolactone) (PCL) as an exemplar polymer, fibers were successfully collected from the same stock solution and spinning parameters for both conventional and cryo- electrospinning methods. Scanning electron microscopy (SEM) images revealed no changes to the fibers’ morphology, which appeared uniform and smooth on the surface (Figure 2A). This indicates that the use of cryogenics did not affect the fiber topography. Furthermore, upon analysis, fiber diameters remained unaffected by the cryo-spinning process when compared to PCL scaffolds (Figure 2B). Whilst excellent for visualizing fiber structure, SEM is limited as a surface imaging technique as it does not allow an accurate assessment of structures that are porous and not entirely flat (i.e., 2D). Incorporating rhodamine into the polymer solution prior to spinning allowed fluorescently tagged fiber scaffolds to be produced that could be successfully imaged by confocal microscopy (Figure 2C). Z-stack images were generated for both fabrication methods, and an assessment of individual slices through this stack demonstrated a visual difference in pore size and porosity. Conventional PCL-electrospun scaffolds were densely packed throughout the z-stack, and pore sizes appeared small. In comparison, the cryo-PCL fibers were sparse when viewed as individual slices, and pore sizes were larger. These two imaging techniques demonstrated that the incorporation of dry ice and post-processing protocol to retain the voids created by the formation of ice crystals between the fibers was successfully achieved.

To better comprehend these structures at a high resolution and in 3D, X-ray computed tomography (X-CT) was applied (Figure 3). In agreement with SEM and confocal microscopy, X-CT images revealed no visual changes in the fibers between the two scaffold groups. A bird’s eye view of the scaffolds suggested no real difference, with both groups displaying a dense network of fibers. Cross-sectional views, however, demonstrated a stark contrast between the two scaffold groups: cryo-PCL fiber scaffolds were evidently thicker, with greater pore sizes and overall porosity (Figure 3B). These visual findings were supported from the structural data extrapolated from reconstructed and binarized TXM files and processing with BoneJ in ImageJ (Table 1). The incorporation of dry ice resulted in significant increases in pore size (+258%), porosity (+130%), and scaffold thickness (+256%), as well as no changes in fiber diameter, when compared to PCL scaffolds. These findings are in agreement with Simonet et al. [24], who similarly reported a significant increase in scaffold thickness and inter-fiber spacing, with no change in fiber diameter for cryo-spun poly(lactic-co-glycolide) acid (PLGA) and poly(ester urethane) (PEU). It is worth noting that this principal paper was published in 2007, and more recent technological advances, such as the use of high-resolution X-CT, means greater accuracy in the data extrapolated can now be achieved.

We have previously reported the structural properties of a human decellularized trabecular meshwork (TM) using X-CT and the same data processing techniques, which allows a direct comparison of these scaffolds [30]. This revealed cryo-PCL scaffolds to be superior in their mimicry of the TM tissue compared to conventional PCL scaffolds, with a similar pore size 8.5 ± 2.0 µm (TM = 8.3 ± 2.8 µm (juxtacanalicular region)) and porosity 91.9 ± 3.4% (TM = 85.0 ± 1.0%). Cryo-PCL scaffolds were also thicker (76.9 ± 10.8 µm) than PCL scaffolds and therefore closer in the mimicry of TM tissue thickness (103 ± 11 µm); a longer spin time should increase the thickness of the cryo-PCL and improve this aspect of recapitulation.

These notable differences in scaffold structure are likely to impact their mechanical properties. As such, tensile testing of both scaffold types was undertaken, and their properties analyzed (Figure 4). Cryo-PCL scaffolds resulted in a six-fold reduction in both Young’s modulus (*p* < 0.0001) and yield stress (*p* < 0.0001) compared to PCL scaffolds. This is a direct consequence of cryo-PCL scaffolds possessing a more open structure, with a greater inter-fiber spacing resulting in a highly porous structure. These observations are in agreement with the literature, where the incorporation of dry ice during electrospun fiber collection resulted in considerably weaker structures [24,36,37]. Tian et al. [37], for example, reported a two-fold reduction in Young’s modulus from 18.34 ± 6.06 MPa to 8.97 ± 1.00 MPa when cryo-spinning a solution of PCL in glacial acid.

A study by Camras et al. reports human cellularized TM to have a tensile stiffness of 51.5 ± 13.6 MPa [38], which is greater than both electrospun scaffolds in this study. Due to the presence of cells, it is difficult to perform a direct comparison with real confidence, and future studies should aim to compare mechanical properties of decellularized TM as cellular content is known to influence strength and stiffness [39,40]. Furthermore, PCL was used as an exemplary polymer with a molecular weight (M_n_) of 70,000 g/mol, in which case, a higher molecular weight [37] or other biocompatible polymers [24,36] may provide more appropriate mechanical properties. Silk, for example, was cryo-spun to create an in vitro mucosal model and achieved a stiffness of 75 MPa [36]. Yet, it is worth noting that a scaffold that is also too strong compared to the native tissue may cause unfavorable effects in low-load bearing tissues like the TM, such as the initiation of a fibrotic response [41]. However, providing the materials are within the biological range and the scaffold supports load, is functional, and encourages integration with the surrounding tissue(s), whilst also undergoing biodegradation at a rate that matches that of new tissue formation to ensure smooth transition of load, then the choice of material remains quite broad [21,42]. Specific to the TM, what rate of degradation is required to match regeneration of tissue is, to the Authors’ knowledge, currently not known and would require a longer-term study in an appropriate environment (e.g., ex vivo TM model [43]).

The assessment of cell attachment to each scaffold was investigated using a transformed human TM cell line (NTM_5_). The aim was to determine whether the change in structure affected how many cells were able to attach within 4 h of seeding. This is based on a published study recognizing that the number of cells seeded is not necessarily the number of cells that attach and therefore grow on the scaffold [44]. In this study, a density of 60,000 cells/cm^2^ was seeded onto PCL and cryo-PCL scaffolds and cultured for a maximum of 4 h. The percentage of cells present on the scaffold, floating in the media, and on the base of the well were calculated using PicoGreen assay (Figure 5). Architecture was deemed to have no effect on cell attachment with both scaffolds supporting a near-identical percentage of cells: 68.2 ± 3.6% for PCL and 68.0 ± 3.4% for cryo-PCL. This equated to 40,800 cells adhering to the scaffolds. This similarity was expected because both scaffolds were made from PCL; therefore, the surface chemistry was the same. Furthermore, scaffolds were spun for the same length of time, meaning the densities of fibers deposited should be equivalent. Whilst cryo-PCL scaffolds possessed a larger pore size, the packing of fibers remained sufficient to prevent a fall-through of cells to the underside of the structure. The number of non-adherent cells was also evenly split between the media (PCL 13.2 ± 1.3%; cryo-PCL 13.7 ± 1.9%) and well (PCL 18.6 ± 2.8%; cryo-PCL 18.3 ± 2.8%) fractions for both scaffold types.

Infiltration of NTM_5_ cells into the electrospun scaffold structures was measured following seven days in culture. This was determined by the immunocytochemical staining of cells and by determining their position to the outer surface of rhodamine-stained fibers (Figure 6). Cells appeared small and circular in shape, which is typical of their phenotype [45] and suggestive of a hospitable environment, though proliferation was not studied at this time. Orthogonal XZ and YZ views of scanned samples were used to quantify cell infiltration by tracking DAPI-stained cell nuclei in the z-direction through the rhodamine-stained fibers. NTM_5_ cells seeded on the cryo-PCL scaffolds infiltrated significantly deeper within the structure, reaching depths of 37.39 ± 6.13 µm (62.31 ± 10.21% scaffold thickness; *p* < 0.0001) compared to cells grown on conventional PCL scaffolds, which penetrated a mere 3.41 ± 1.17 µm (11.34 ± 3.90% scaffold thickness) from the outer surface. These findings are, however, expected because the increased pore size of the cryo-PCL scaffold and overall increased porosity provides the cells with an environment more accommodating for their migration into the structure. Leong et al. similarly demonstrated enhanced infiltration of 3T3/NIF fibroblasts within cryo-spun poly(lactic acid) scaffolds, with cells penetrating up to 80% of the scaffold thickness after 14 days culture [25]. Therefore, it suggests that cryo-electrospun scaffolds could support three-dimensional cell growth, which may promote a more in vivo-like environment for modeling of the TM (and other tissues) over longer periods of in vitro culture.

## 5. Conclusions

The effects of pore size and porosity on cell infiltration into electrospun fiber scaffolds were evaluated. Scaffolds of differing pore sizes and porosities were created by simply altering the method of fiber collection during electrospinning, whilst keeping all other parameters constant. The incorporation of dry ice inside the collecting mandrel encouraged ice crystal formation, which created larger inter-fiber spacing that was retained following sublimation. The increased pore size, porosity, and overall scaffold thickness were all closer to mimicking the structure of human trabecular meshwork compared to conventional PCL fiber scaffolds. Furthermore, these cryo-PCL scaffolds supported the attachment and infiltration of a healthy, human trabecular meshwork cell line, which reached 62.31 ± 10.21% of the total scaffold thickness.

This study demonstrates the utility of cryo-electrospun scaffolds as a mimic of trabecular meshwork and its potential application as a 3D in vitro model to support further study on this tissue and its role in glaucoma research.

## Figures and Tables

**Table 1:** **Cryo-spinning increases scaffold porosity, pore size, and thickness compared to conventional electrospinning.** Structural dimensions of electrospun poly(ε-caprolactone) (PCL) and cryo-electrospun PCL (cryo-PCL). Data for scaffold porosity, pore diameter, and thickness measured using BoneJ plugin for ImageJ from X-ray computed tomography images; presented as mean ± standard deviation with unpaired *t*-test statistical analysis (*p* < 0.05).

	PCL	Cryo-PCL
Pore Diameter (µm)	3.3 ± 0.8	8.5 ± 2.0 ^a^
Porosity (%)	70.9 ± 3.8	91.9 ± 3.4 ^b^
Scaffold Thickness (µm)	30.0 ± 2.9	76.9 ± 10.8 ^c^

^a–c^ Significance level for cryo-PCL compared to PCL within row (*p* < 0.05).

## Data Availability

All data can be found using the following https://doi.org/10.17638/datacat.liverpool.ac.uk/2447 (accessed 31 August 2023).
